# Supplementary material for: Novel Driver Strength Index highlights important cancer genes in TCGA PanCanAtlas patients
Source: PeerJ. 2022 Aug 11;10:e13860. doi: 10.7717/peerj.13860 (PMC9375969; doi:10.7717/peerj.13860)
Supplement: Supplemental Information 2 [file peerj-10-13860-s002.zip › COHORTS/genes plots/age/2021_8_16_14_9_distribution_cohorts_30-34.pdf]

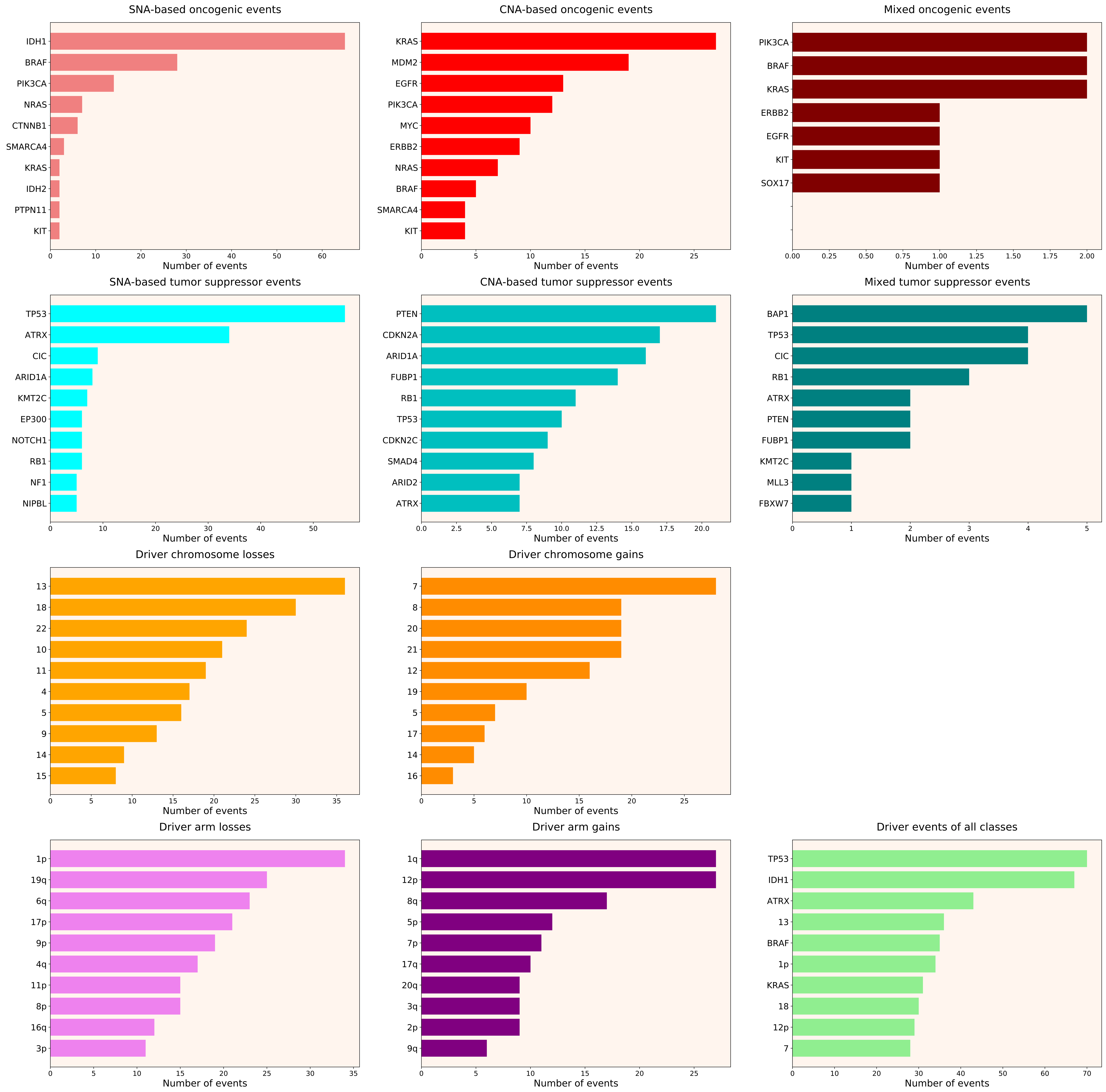
SNA-based oncogenic events
IDH1
BRAF
PIK3CA
NRAS
CTNNB1
SMARCA4
KRAS
IDH2
PTPN11
KIT
0 10 20 30 40 50 60
Number of events
CNA-based oncogenic events
KRAS
MDM2
EGFR
PIK3CA
MYC
ERBB2
NRAS
BRAF
SMARCA4
KIT
0 5 10 15 20 25
Number of events
Mixed oncogenic events
PIK3CA
BRAF
KRAS
ERBB2
EGFR
KIT
SOX17
0.00 0.25 0.50 0.75 1.00 1.25 1.50 1.75 2.00
Number of events
SNA-based tumor suppressor events
TP53
ATRX
CIC
ARID1A
KMT2C
EP300
NOTCH1
RB1
NF1
NIPBL
0 10 20 30 40 50
Number of events
CNA-based tumor suppressor events
PTEN
CDKN2A
ARID1A
FUBP1
RB1
TP53
CDKN2C
SMAD4
ARID2
ATRX
0.0 2.5 5.0 7.5 10.0 12.5 15.0 17.5 20.0
Number of events
Mixed tumor suppressor events
BAP1
TP53
CIC
RB1
ATRX
PTEN
FUBP1
KMT2C
MLL3
FBXW7
0 1 2 3 4 5
Number of events
Driver chromosome losses
13
18
22
10
11
4
5
9
14
15
0 5 10 15 20 25 30 35
Number of events
Driver chromosome gains
7
8
20
21
12
19
5
17
14
16
0 5 10 15 20 25
Number of events
Driver arm losses
1p
19q
6q
17p
9p
4q
11p
8p
16q
3p
0 5 10 15 20 25 30 35
Number of events
Driver arm gains
1q
12p
8q
5p
7p
17q
20q
3q
2p
9q
0 5 10 15 20 25
Number of events
Driver events of all classes
TP53
IDH1
ATRX
13
BRAF
1p
KRAS
18
12p
7
0 10 20 30 40 50 60 70
Number of events
